# Whole genome sequencing of extended-spectrum β-lactamase producing *Klebsiella pneumoniae* isolated from a patient in Lebanon

**DOI:** 10.3389/fcimb.2015.00032

**Published:** 2015-04-08

**Authors:** Sima Tokajian, Jonathan A. Eisen, Guillaume Jospin, Anna Farra, David A. Coil

**Affiliations:** ^1^Department of Natural Sciences, School of Arts and Sciences, Lebanese American UniversityByblos, Lebanon; ^2^University of California Davis Genome CenterDavis, CA, USA; ^3^School of Medicine, Lebanese American UniversityByblos, Lebanon

**Keywords:** ESBL, *Klebsiella pneumoniae*, whole genome sequencing, CTX-M-15, SHV-11

## Abstract

**Objective:** The emergence of extended-spectrum β-lactamase (ESBL)-producing bacteria is now a critical concern. The ESBL-producing *Klebsiella pneumoniae* constitutes one of the most common multidrug-resistant (MDR) groups of gram-negative bacteria involved in nosocomial infections worldwide. In this study we report on the molecular characterization through whole genome sequencing of an ESBL-producing *K. pneumoniae* strain, LAU-KP1, isolated from a stool sample from a patient admitted for a gastrointestinal procedure/surgery at the Lebanese Amrican University Medical Center-Rizk Hospital (LAUMCRH) in Lebanon.

**Methods:** Illumina paired-end libraries were prepared and sequenced, which resulted in 4,220,969 high-quality reads. All sequence processing and assembly were performed using the A5 assembly pipeline.

**Results:** The initial assembly produced 86 contigs, for which no scaffolding was obtained. The final collection of contigs was submitted to GenBank. The final draft genome sequence consists of a combined 5,632,663 bases with 57% G+C content. Automated annotation was performed using the RAST annotation server. Sequencing analysis revealed that the isolate harbored different β-lactamase genes, including *bla*_oxa−1_, *bla*_CTX−M−15_, *bla*_SHV−11_, and *bla*_TEM−1b_. The isolate was also characterized by the concomitant presence of other resistance determinants most notably *acc(6′)-lb-cr* and *qnrb1*. The entire plasmid content was also investigated and revealed homology with four major plasmids pKPN-IT, pBS512_2, pRSF1010_SL1344, and pKPN3.

**Conclusions:** The potential role of *K. pneumonia* as a reservoir for ESBL genes and other resistance determinants is along with the presence of key factors that favor the spread of antimicrobial resistance a clear cause of concern and the problem that Carbapenem-non-susceptible ESBL isolates are posing in hospitals should be reconsidered through systematic exploration and molecular characterization.

## Introduction

Extended-spectrum β-lactamases (ESBL) are enzymes produced by many bacterial species as a means for defense against β-lactam drugs with the genes encoding for those enzymes being mainly located on mobile genetic elements (Pfeifer et al., 2010). ESBL enzymes confer antimicrobial resistance to a wide spectrum of β-lactams such as penicillins, aztreonam, as well as first, second, and third generation cephalosporins (Jacoby and Medeiros, 1991; Bradford, 2001). β-lactamases are widespread and were detected in a wide range of *Enterobacteriaceae* (Bradford, 2001) predominantly in *Escherichia coli*, and *Klebsiella pneumoniae*, as well as in other non-enteric microorganisms, such as *Pseudomonas aeruginosa, Acinetobacter baumanni* (Naas et al., 1999; Spanu et al., 2002; Goossens and Grabein, 2005; Paterson and Bonomo, 2005), and *Capnocytophaga ochracea* (Rosenau et al., 2000). In addition, *Shigella* species have been increasingly reported to be resistant to β-lactams including third-generation cephalosporins (Levesque et al., 1995).

*K. pneumoniae* is an important human pathogen causing nosocomial infections (Podschun and Ullmann, 1998). ESBL-producing *K. pneumoniae* is one of the most common multidrug-resistant (MDR) groups of gram-negative bacteria worldwide (Breurec et al., 2013). Infections caused by ESBL-producing *K. pneumoniae* are often associated with the urinary and respiratory tracts along with epidemic clones causing outbreaks in intensive care units (Peirana et al., 2012).

There are four molecular classes of β-lactamases. Classes A, C, and D. β-lactamases possess an active-site serine, while class B β-lactamases are metalloenzymes requiring Zn molecule for their activity (Ambler et al., 1991; Bush et al., 1995). CTX-M is a dominant ESBL family in *K. pneumoniae* strains, but TEM and SHV enzymes in addition to members of the three classes of β-lactamases (B, C, D) are also common (Breurec et al., 2013). Carbapenems are usually used for the treatment of infections caused by ESBL producing *Enterobacteriaceae*. Increasing rates of ESBL-producing isolates has led to the overuse of carbapenems creating a pressure and triggering resistance. Resistance to carbapenems may be associated with the production of carbapenemases including class A KPCs, class B metallo-β-lactamases (VIM, IMP, or NDM-1) or the class D OXA-type enzymes (Queenan and Bush, 2007; Matar et al., 2008; Nordmann et al., 2009).

The aim of this study is to characterize an ESBL-producing *K. pneumoniae* isolated from a stool sample from a patient admitted for a gastrointestinal procedure/surgery in a Lebanese hospital using whole genome sequencing (WGS).

## Materials and methods

### Ethical approval

The University's Institutional Review Board approved the study (UMCRH.AF.12/Dec/2012).

### Study design and bacterial isolate

Samples were collected from the University Medical Center-Rizk Hospital (UMC-RH). All patients included in the study were screened for the presence of ESBL-producing *Enterobacteriaceae* by means of three consecutive rectal swab or stool cultures, as per regular screening for ESBL detection. The first fecal sample was collected at the same time as the other preoperative tests. The second and third samples were collected later, latest day 2-post surgery. *K. pneumoniae* LAU-KP1 sequenced in this study was isolated from a 62-year-old woman working as practitioner nurse or nurse's assistant. She was admitted for radical resection of an abdominal tumor and repair of abdominal hernia. The patient had other than her cancer, recurrent urinary tract infections. She had been using amoxicillin-clavulanate, metronidazol, and cefriaxon in the 3 months prior to presentation. She had been admitted to hospital in the year prior to surgery, but was not known to be a carrier of multidrug resistant organism and no one in her household was known to be a carrier of multidrug resistant organisms.

### Screening for ESBL

The standard disk diffusion method with combined discs (ceftazidime/clavulanate and cefotaxime/clavulanate) was used for screening. The standard *E*-test method using double sided strips containing on one side ceftazidime or cefotaxime and on the other the same antibiotic combined with clavulanate was used to assess for screening and confirming ESBL production in all strains according to the recommendations of the CLSI. *E. coli* ATCC25922 and *K. pneumoniae* ATCC700603 were used as the quality control strains.

### Antimicrobial testing

Antimicrobial susceptibility test by the disk diffusion method was performed to determine the resistance patterns of the isolates to 17 antibiotics: cefotaxime, ceftriaxone, ceftazidime, aztreonam, ceftazidime/clavulanate, cefotaxime/clavulanate, cephalothin, cefuroxime sodium, cefepime, gentamicin, amikacin, ciprofloxacin, tetracycline, ampicillin, amoxicillin/clavulanate, piperacillin, piperacillin/tazobactam, imipenem, meropenem, ertapenem, and sulfamethoxazole/trimethoprim [Oxoid, England; disc contents according to Clinical and Laboratory Standards Institute (CLSI) guidelines]. All antimicrobial testing was performed on Mueller-Hinton agar by the flooding technique and data interpreted according to the CLSI guidelines.

### DNA isolation and sequencing

Bacterial DNA was extracted using the Nucleospin kit (Macherey-Nagel) and following the manufacturer's instructions.

### Genome sequencing

Genomic DNA (gDNA) was used as input for library preparation using the Illumina TruSeq DNA library preparation kit (Illumina). Bioruptor® NGS was used to sonicate 1 μg of sample DNA (100 μl final sonication volume in TE buffer) for 8 min of 30 s on/off cycles at 4°C. Sheared DNA was used as input for library preparation using the Illumina TruSeq DNA library preparation kit (Illumina). The gDNA was subjected to end-repair, A-tailing, ligation of adaptors including sample-specific barcodes as recommended in the manufacturer's protocol. After ligation of the adaptors, and to reduce the number of fragments smaller than 500 bp, fragments between 500 and 1000 bp were selected using a Pippin Prep™ DNA size selection system (Sage Science). The resulting library was quantified by quantitative PCR in triplicate at 1:1000 and using the Kapa library quantification kit (Kapa Biosystems, Woburn, MA) and as recommended by the manufacturer's protocol. The resultant library size was assessed using an Agilent Bioanalyzer with the High Sensitivity DNA Kit. The library was multiplexed, clustered, and sequenced on an Illumina MiSeq with paired-end 500 cycles protocol to read a length of 250 bp.

### Genome assembly

Genome assembly was performed *de novo* using A5 with default parameters (Tritt et al., 2012). This pipeline automates the processes of data cleaning, error correction, contig assembly, scaffolding and quality control.

### Genome analysis

The assembly was uploaded and annotated using RAST server (http://rast.nmpdr.org). The service identified protein-encoding, rRNA and tRNA genes, assigned functions to the genes, and predicted which subsystems are represented in the genome (Larsen et al., 2012).

The multi-locus sequence type for the isolate was determined from the WGS data. The presence of known acquired resistance genes was determined by mapping the data from the isolate to an online database. The ResFinder web server (www.genomicepidemiology.org) was used to identify acquired antimicrobial resistance genes in the WGS data, using a threshold of 98.00% identity (ID) (Zankari et al., 2012). ResFinder detects the presence of whole resistance genes, but not functional integrity and expression or resistance due to acquired variation in housekeeping genes. Based on the ResFinder results, a predicted phenotype was determined using phenotypes from original published studies of the genes found.

A *K. pneumoniae* concatenated marker gene maximum-likelihood tree was constructed using all available assembled *K. pneumoniae* genomes on the NCBI ftp server. The genomes were first processed with PhyloSift (Darling et al., 2014) using –besthit and –isolate options to get alignments to concatenated conserved housekeeping genes. The tree was made using FastTree (Price et al., 2010). The tree was edited and visualized using Dendroscope (Huson and Scornavacca, 2012). Clades with zero branch lengths were collapsed to a single representative. For visualization of LAU-KP1, the assembly was aligned against the reference strain *K. pneumoniae* Ecl8's (GCF_000315385) using progressiveMauve (Darling et al., 2010). Ring representation was obtained using BRIG 0.95 (Alikhan et al., 2011). Ecl8 is the *K. pneumoniae* isolate which was closest to LAU-KP1 in the concatenated marker gene tree that had a complete genome.

### Nucleotide sequence accession number

The draft sequence of the *K. pneumoniae* LAU-KP1 have been deposited at DDBJ/EMBL/GenBank under the accession no. AYQE00000000.1.

## Results and discussion

To the best of our knowledge we report for the first time the isolation of a CTX-M-15, SHV-11, KPC-producing *K. pneumoniae* belonging to ST336 in Lebanon. This clone belongs to CG17 and was isolated from a 62-year-old woman working as practitioner nurse or nurse's assistant. She was admitted for radical resection of an abdominal tumor and repair of abdominal hernia. The patient had other than her cancer, recurrent urinary tract infections.

In *K. pneumoniae* MLST is based on genetic variation in seven housekeeping genes (*rpoB, gapA, mdh, pgi, phoE, infB*, and *tonB*) (Diancourt et al., 2005). ST allele profile of ST336 is: 2-1-1-1-72-4-4, while ST258, being the most common KPC-producing isolate (Woodford et al., 2011), is: 3-3-1-1-1-1-79 (http://www.pasteur.fr/mlst). Screening patients for rectal carriage by Baraniak et al. (2013) revealed the prevalence of CG17 in France, highlighting the role of human mobility in spreading MDR *K. pneumoniae* clones.

LAU-KP1 was found to be resistant to the following drugs: ciprofloxacin, cefotaxime, aztreonam, and ceftazidime. It was intermediate to amoxicillin clavulanic acid, and sensitive to imipenem and gentamycin. Analysis of the data extracted from the draft genome sequence however, revealed that LAU-KP1 was characterized by the presence of multiple resistance determinants, most notably was the concomitant presence of *acc(6′*)lb-cr, *qnr*, SHV-11, and CTX-M-15. CTX-M-15 detection was consistent with the recent predominance of CTX-M and CTX-M-15 in particular (Rossolini et al., 2008), which additionally was found to be more common in *K. pneumoniae* than in *E. coli* in rehabilitation units (Baraniak et al., 2013). Rodrigues et al. (2014) reported recently that the increase in the incidence of CTX-M-15 and diverse SHV ESBL types observed in a Portuguese hospital was associated with the increase of MDR *K. pneumoniae* epidemic clones (ST15, ST147, ST336). Harbouring the *qnr*B1 (quinolone resistance) was another important finding that additionally distinguished LAU-KP1. Previously Breurec et al. (2013) revealed that *qnr*B was predominant among strains from Africa while *qnr*S was mainly detected in strains from Vietnam.

Resistance to carbapenems involves multiple mechanisms including alterations in outer membrane permeability mediated by the loss of porins, upregulation of efflux systems along with hyper production of AmpC β-lactamases or more commonly the production of carbapenemases (Chen et al., 2014). Class A carbapenemases include the *K. pneumoniae* carbapenemase (KPC). These enzymes hydrolyze all β-lactams including monobactams (El-Herte et al., 2012). Carbapenem resistant *Enterobacteriaceae have* spread in Northeastern USA and to other countries including Argentina, Greece, Italy, and China (Nordmann and Poirel, 2014). Transmission of the KPC gene can be mediated through horizontal gene transfer on mobile elements including transposons and plasmids (Munoz-Price and Quinn, 2009). Spread of the KPC-producing *K. pneumonia* has been linked to a major multi locus sequence type (MLST-ST) ST258 and its variants (Cuzon et al., 2010; Chen et al., 2014). LAU-KP1 harbored INCFII*_K_* plasmids and DNA comparison showed the presence of sequences with high homology to plasmid pKPN-IT and pKPN3. This was consistent with the finding that the dissemination of CTX-M-15 was primarily associated with IncFII_K_ plasmid in *K. pneumoniae* isolates (Coelhoa et al., 2010). Earlier Garcia-Fernandez et al. (2012) detected in *K. pneumoniae* ST258 IncFII_K_-FIB like plasmid and was named as pKPN-IT. This plasmid was highly related to plasmid pKPN3. The plasmid pKPN-IT was shown to also acquire Fec-like iron (III) dicitrate transport system and a class I integron carrying trimethoprim resistance, both of which were also detected in LAU-KP1.

β-lactamase class C, the presence of which renders bacteria to become resistant to most β-lactams including cephamycins and β-lactam/β-lactamase combinations (Doi and Paterson, 2007) was and along with macrolide-specific efflux pump MacAB-TolC important factors increasing the resistance of LAU-KP1. Periplasmic membrane fusion proteins (MFPs) are essential components of the type I protein secretion systems and drug efflux pumps in Gram-negative bacteria (Tikhonova et al., 2007). MacA is a peripheral membrane protein of the MFP family and a component of the MacAB macrolide efflux transporter complex. MacB on the other hand, is an integral membrane protein. Similar to other MFP-dependent transporters from *E. coli*, MacAB requires the outer membrane channel TolC for its function (Kobayashi et al., 2001; Tikhonova et al., 2007).

Additionally, a key feature of these MDR efflux systems is their ability to extrude a broad spectrum of substrates, including various antimicrobial agents (Bambeke et al., 2000; Putman et al., 2000; Poole, 2001). One important family of drug transporters that contribute to multidrug resistance (MDR) in gram-negative bacteria are the resistance-nodulation-cell division (RND) efflux systems, which consist of an inner membrane transporter, a periplasmic fusion protein, and an outer membrane protein (Zgurskaya and Nikaido, 2000). Detecting CmeABC operon in LAU-KP1 was an important feature. CmeABC is an energy-dependent efflux system contributing to the intrinsic resistance of *Campylobacter* to diverse antimicrobial agents. Disruption of the CmeABC resulted in MICs that were 8-fold lower for ciprofloxacin, 2-fold for norfloxacin and nalidixic acid, 256-fold for cefotaxime, and 32-fold for ampicillin (Lin et al., 2002).

In *K. pneumoniae* MLST is based on genetic variation in seven housekeeping genes (*rpoB, gapA, mdh, pgi, phoE, infB*, and *tonB*) (Diancourt et al., 2005). ST allele profile of ST336 is: 2-1-1-1-72-4-4, while ST258, being the most common KPC-producing isolate (Woodford et al., 2011), is: 3-3-1-1-1-1-79 (http://www.pasteur.fr/mlst). Screening patients for rectal carriage by Baraniak et al. (2013) revealed the prevalence of CG17 in France, highlighting the role of human mobility in spreading MDR *K. pneumoniae* clones.

**Figure 1:**
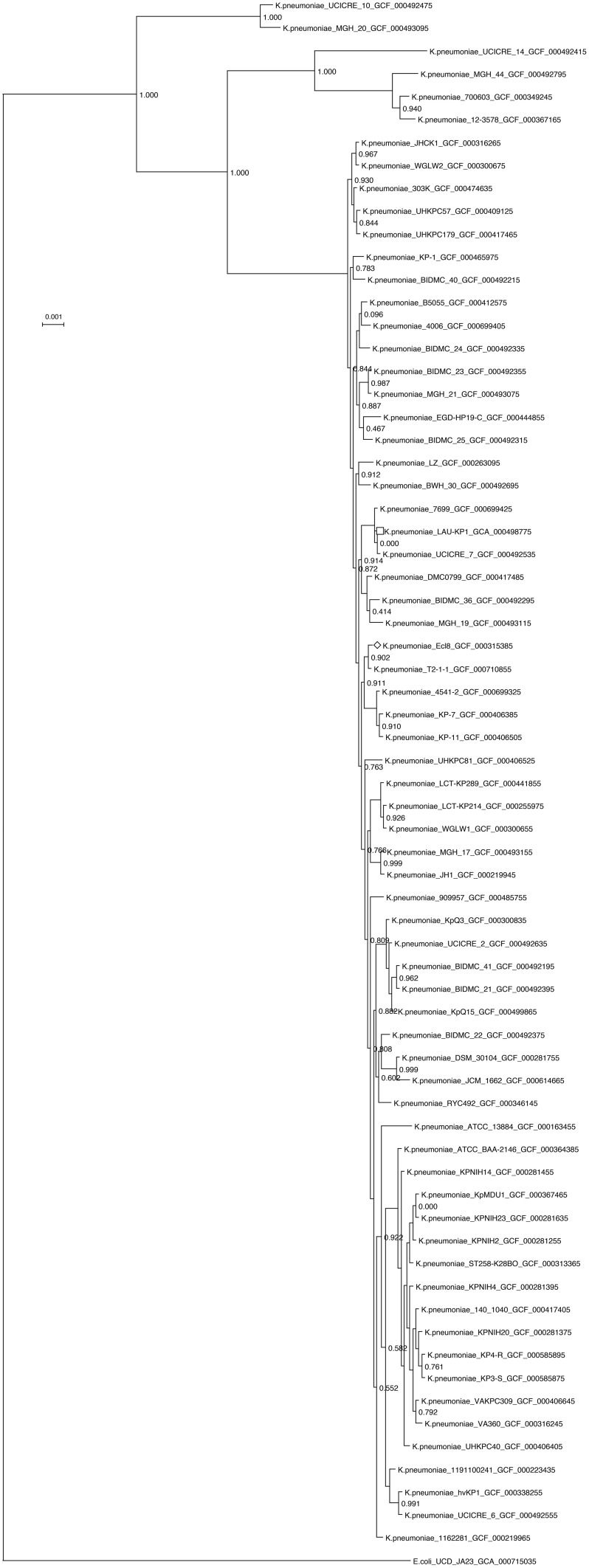
**Phylogenetic tree of all assembled *K. pneumoniae* genomes available on the NCBI ftp server as of 2-11-15, based on a concatenated alignment of 37 conserved genes obtained using PhyloSift (Darling et al., 2014)**. The tree was constructed using default settings in FastTree (Price et al., 2010) and edited and visualized with Dendroscope v.3 (Huson and Scornavacca, 2012). Clades with zero branch lengths were collapsed to a single representative. *K. pneumoniae* LAU-KP1 is indicated by a square, and the reference genome used in Figure 2 is indicated by a diamond.

**Figure 2:**
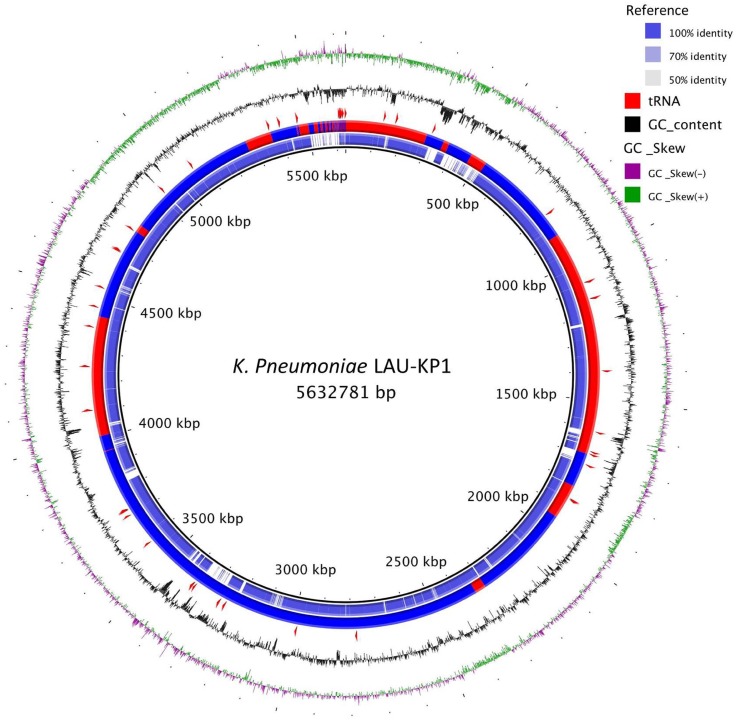
***K. pneumoniae* LAU-KP1 ring representation using BRIG 0.95 (Alikhan et al., 2011)**. The contigs from LAU-KP1 were ordered to match Ecl8's (GCF_000315385) genomic arrangement using progressiveMauve (Darling et al., 2010). Ecl8 is the *K. pneumoniae* isolate which was closest to LAU-KP1 in the tree shown in Figure 1 for which there was a finished genome available. The inner ring shows the percent identity comparing Ecl8 and LAU-KP1. The next ring alternating blue and red sections shows the contig delimitations for LAU-KP1. The next ring shows the location of tRNAs according to Prokka v1.7 (Seemann, 2014). The last two (outer) rings show the GC content and skew.

Furthermore, the phylogenetic analysis of LAU-KP1 showed that it falls within a clade of other known multidrug resistant *K. pneumoniae* strains and demonstrated that there is considerable genomic diversity among the included isolates (Figure 1). Within the closely related isolates was *K. pneumonia* 7699 previously cultured from a perianal swab of a patient admitted to the intensive care unit of the University of Maryland Medical Center (UMMC) in Baltimore, MD (Hazen et al., 2014). The isolate had a 167-kb IncA/C plasmid encoding the FOX-5 β-lactamase, a CARB-2 β-lactamase, other antimicrobial resistance genes, and heavy metal resistance genes. On the other hand, a considerable phylogenetic difference was detected between LAU-Kp1 and *K. pneumoniae* ATCC BAA-2146, which is the first U.S. isolate found encoding the gene for the broad-spectrum and carbapenem-active metallo-β-lactamase NDM-1 (Hudson et al., 2014). Additionally, we mapped our contigs to the phylogenetically closest *K. pneumoniae* isolate with a complete genome, Ecl8, in order to visualize the genomic organization (Figure 2).

The detection of such a multidrug resistant strain of *K. pneumoniae* emphasizes the potential for transmission of highly resistant gram-negative pathogens that are difficult to treat. This merits more detailed future studies directed toward characterization of molecular mechanisms, environmental factors, and selection pressures promoting the spread of such strains. Detection of IncFII_K_ a virulence plasmid (Dolejska et al., 2013) known to evolve quickly by replicon diversification and acquisition of antibiotic resistance traits (Coelhoa et al., 2010; Carattoli, 2013), increases the potential role of *K. pneumoniae* as a reservoir for ESBL genes and other resistance determinants. The spread of antimicrobial resistance in this region could be attributed to different factors including: uncontrolled consumption of antimicrobial agents through self-medication, inappropriate antibiotic prescription, the substandard quality of some drugs, and a lack of effective measures to prevent nosocomial infections. The virtual lack of physical barriers between the community and hospital settings in the countries in this region and the increasing pressure from war immigrants along with poor living conditions may be also important contributory factors. High-resolution genotyping studies of isolates collected over larger spatial and temporal scales will be necessary to decipher the dynamics of the emergence of MDR *K. pneumoniae* clonal groups.

Therefore, we urgently need well-designed epidemiological and molecular studies to understand the dynamics of transmission, risk factors, and reservoirs for *K. pneumoniae*. This will provide a better insight into the emergence and spread of these multidrug resistant strains and hopefully lead to information essential in preventing infections and limiting the spread of such organisms.

### Conflict of interest statement

The authors declare that the research was conducted in the absence of any commercial or financial relationships that could be construed as a potential conflict of interest.
